# Estimated Risk for Altered Fetal Growth Resulting from Exposure to Fine Particles during Pregnancy: An Epidemiologic Prospective Cohort Study in Poland

**DOI:** 10.1289/ehp.7065

**Published:** 2004-06-21

**Authors:** Wieslaw Jedrychowski, Ivona Bendkowska, Elzbieta Flak, Agnieszka Penar, Ryszard Jacek, Irena Kaim, John D. Spengler, David Camann, Frederica P. Perera

**Affiliations:** ^1^Epidemiology and Preventive Medicine, Medical College, Jagiellonian University, Krakow, Poland; ^2^Center for Children’s Environmental Health, Mailman School of Public Health, Columbia University, New York, New York, USA; ^3^Obstetrics and Gynecology, Medical College, Jagiellonian University, Krakow, Poland; ^4^Department of Environmental Health, School of Public Health, Harvard University, Boston, Massachusetts, USA; ^5^Southwest Research Institute, San Antonio, Texas, USA

**Keywords:** air pollutants, cohort study, fetal growth, pregnancy, prenatal exposure

## Abstract

The purpose of this study was to estimate exposure of pregnant women in Poland to fine particulate matter [≤2.5 μm in diameter (PM_2.5_)] and to assess its effect on the birth outcomes. The cohort consisted of 362 pregnant women who gave birth between 34 and 43 weeks of gestation. The enrollment included only nonsmoking women with singleton pregnancies, 18–35 years of age, who were free from chronic diseases such as diabetes and hypertension. PM_2.5_ was measured by personal air monitoring over 48 hr during the second trimester of pregnancy. All assessed birth effects were adjusted in multiple linear regression models for potential confounding factors such as the size of mother (maternal height, prepregnancy weight), parity, sex of child, gestational age, season of birth, and self-reported environmental tobacco smoke (ETS). The regression model explained 35% of the variability in birth weight (β = −200.8, *p* = 0.03), and both regression coefficients for PM_2.5_ and birth length (β = −1.44, *p* = 0.01) and head circumference (HC; β = −0.73, *p* = 0.02) were significant as well. In all regression models, the effect of ETS was insignificant. Predicted reduction in birth weight at an increase of exposure from 10 to 50 μg/m^3^ was 140.3 g. The corresponding predicted reduction of birth length would be 1.0 cm, and of HC, 0.5 cm. The study provides new and convincing epidemiologic evidence that high personal exposure to fine particles is associated with adverse effects on the developing fetus. These results indicate the need to reduce ambient fine particulate concentrations. However, further research should establish possible biologic mechanisms explaining the observed relationship.

A large body of evidence demonstrates that, in addition to parental smoking (Bosley et al. 1981; Jedrychowski et al. 1977; Kallen 1997a, 1997b; Lowe 1959; Martinez et al. 1994; Miller et al. 1976; Savitz et al. 1991; Shaw et al. 1996; Simpson 1957; Underwood et al. 1967; Wasserman et al. 1996; Wyszynski et al. 1997) and environmental tobacco smoke (ETS) [Jedrychowski and Flak 1996; Mainous and Hueston 1994; Martin and Bracken 1986; National Research Council 1986; Ogawa et al. 1991; Perera et al. 2004; Rubin et al. 1986; U.S. Environmental Protection Agency (EPA) 1992; Windham et al. 1999), outdoor and indoor air pollutants may increase the risk of adverse birth outcomes, including low birth weight (LBW), premature births, and intrauterine growth retardation (IUGR) (Antipenko and Kogut 1993; Axelsson and Molin 1988; Bhopal et al. 1994, 1998; Bobak 2000; Bobak and Leon 1999; Dejmek et al. 1999, 2000; Kavlock et al. 1979; Landgren 1996; Lin et al. 2001; Longo 1997; Loomis et al. 1999; Norska-Borowka and Bursa 1993; Pereira et al. 1998; Perera et al. 1998, 2003; Ritz and Yu 1999; Ritz et al. 2000; Singh 1988; Smrcka and Leznarova 1998; Sram 1999; Tabacova and Balabaeva 1993; Wang et al. 1997; Woodruff et al. 1997; Xu et al. 1995). Despite the large number of studies dealing with air pollutants and birth outcomes, the evidence for a causal association remains still weak. Other studies comparing areas with wide ranges of exposure are needed to show the evidence for small effects.

It is assumed that areas in and around the home are important sources of chemical exposures for pregnant women, fetuses, and newborns. Toxic chemicals particularly may be present in proximity to industrial complexes and hazardous waste sites, as well as deriving from local combustion sources such as cars, trucks, and bus routes. The home can also be subject to contamination by particulate matter (PM) and compounds such as nitrogen dioxide, sulfur dioxide, carbon monoxide, and polycyclic aromatic hydrocarbons (PAHs).

Reproductive epidemiology provides evidence that fetuses and infants are likely to be significantly more sensitive to a variety of environmental toxicants than are adults. They are more sensitive because of differential exposure, physiologic immaturity, and a longer lifetime over which disease initiated in the early life can develop. Newborns and young children are especially vulnerable to the toxic effects of ETS, PAHs, PM, nitrosamines, pesticides, polychlorinated biphenyls, metals, and radiation (Perera et al. 2002).

The major difficulty in studying birth outcomes associated with air pollution lies in assessing exposure. Previous studies have attempted to quantify the concentration of outdoor air pollutants in the residence area such as total suspended particulates (TSP), particulate matter ≤10 μm in diameter (PM_10_), SO_2_, or CO and assign exposure values to the study subjects or use the area-based exposures to approximate individual exposures. Most prior studies, especially ecologic ones, did not consider important confounding factors such as maternal height and prepregnancy weight, smoking status, or occupational exposure.

The purpose of the present study was to estimate the exposure of pregnant women in Poland to potentially hazardous fine PM [≤2.5 μm in diameter (PM_2.5_)] and to assess its effects on the birth outcomes [weight, length, and head circumference (HC) at birth]. To avoid potential methodologic limitations of previous studies regarding the assessment of exposure, we included assessment of personal individual exposure to fine particulate pollutants from all potential sources indoors and outdoors. In the analysis of the association between air pollutants and birth outcomes, we also considered important confounders such as maternal anthropometric characteristics, parity, sex of child, gestational age, and birth season.

## Materials and Methods

The design of this cohort prospective study and the detailed selection of the population have been described previously (Jedrychowski et al. 2003). Briefly, this study is part of an ongoing comparative longitudinal investigation of the health impact of prenatal exposure to outdoor/indoor air pollution on infants and children being conducted in New York City and Krakow. The ethics committee of the Jagiellonian University approved the study.

We analyzed data from 362 women who gave birth between 34 and 42 weeks of gestation from January 2001 through March 2003. Women attending ambulatory prenatal clinics in the first and second trimesters of pregnancy were eligible for the study. The enrollment included only nonsmoking women with singleton pregnancies who were 18–35 years of age and were free from chronic diseases such as diabetes and hypertension. Recruited women were interviewed and given a description of the study and requirements for participation in the project. Each subject was given a detailed questionnaire at entry to the study and in the third trimester to solicit information on demographic data, house characteristics, date of the last menstrual period (LMP), medical and reproductive history, occupational hazards, alcohol consumption, and smoking practices of others present in the home. After participating women had given birth, maternal and child hospital records were reviewed to obtain data on complications of delivery. Weight, length, and HC at birth and Apgar score at 1 and 5 min were recorded for all infants. Gestational age at birth was defined as the interval between the last day of the mother’s LMP and the date of birth.

### Dosimetry of prenatal personal exposure to fine particles.

During the second trimester, a member of the air monitoring staff instructed the women in the use of the personal monitor, which is lightweight and quiet and is worn in a backpack. The women were asked to wear the monitor during daytime hours for 2 consecutive days and to place the monitor near the bed at night. During the morning of the second day, the air monitoring staff person and interviewer visited the women’s homes to change the battery pack and administer the full questionnaire. They also checked to see that the monitor had been running continuously and that no technical or operating failures had occurred. A staff member returned to the women’s homes on the morning of the third day to pick up the equipment.

A personal environmental monitoring sampler (PEMS) was used to measure particle mass. The PEMS is designed to achieve the particle target size of ≤2.5 μm at a flow rate of 4.0 L/min with an array of 10 impactor nozzles. Flow rates were calibrated (with filters in place) before the monitoring and were checked again with a change of the battery pack on the second day and at the conclusion of the monitoring. Pumps operated continuously at 2 L/min over the 48-hr period. To modify the sampler to achieve the 2.5-μm size cutoff at 2 L/min, five of the nozzles were blocked. Particles were collected on Teflon membrane filter (37 mm Teflo; SKC, Inc., Eighty Four, PA, USA). The combination of low pressure drop (permitting use of a low-power sampling pump), low hygroscopicity (minimizing bound water interference in mass measurements), and low trace element background (improving analytical sensitivity) of these filters makes them highly appropriate for personal particle sampling. Dust air samples were analyzed by J.D.S. and his staff.

### Statistical methods.

The main birth outcomes were birth weight, length, and HC at birth, and association with exposure was examined by univariate and multivariate models. We constructed several models where exposure to PM_2.5_ was treated as continuous and dichotomous variables. First, crude effects were estimated, and subsequently they were adjusted to confounders. All maternal factors included in multivariate analyses were related to birth outcomes in bivariate analysis. In the final statistical analysis, we assessed the effect of PM_2.5_ and ETS exposure on the birth out-comes by multiple linear regression, controlling for potential confounders (parity, height of mother, prepregnancy weight, sex of infant, gestational age, and season of birth). We tested seasons of year for confounding because of their association with exposure and their potential association with fetal growth. Season of birth was introduced in the regression models as a dummy variable, with summer defined as the reference level. Because the distribution of the air pollutants in question was skewed, the PM_2.5_ values were log transformed before entry into the regression models. Because the outliers of exposure to PM_2.5_ did not change the regression estimates, they were not removed from the analysis. The ETS variable was categorized as follows: 1, no exposure; 2, exposed to ≤10 cigarettes smoked daily at home; 3, exposed to 11–20 cigarettes smoked daily at home; 4, exposed to > 20 cigarettes smoked daily at home. In all statistical analyses, the significance level was set at *p* < 0.05.

For testing the functional relationship between PM_2.5_ and birth outcomes, we used generalized additive models in S-Plus (Mathsoft Inc., Seattle, WA, USA; Mathsoft 1999), including gestational age, sex of child, parity, height and prepregnancy weight of mother, and the delivery season. The analysis showed that the relationship between PM_2.5_ and birth length did not differ from the linear relationship established by the linear multivariate regression model. The reduction of residual sum of squares from 1978.73 for linear fit to 1955.09 was not significant (*p* = 0.237). The corresponding estimates for birth weight and HC were similar.

## Results

Analysis of personal air samples from the 362 pregnant women enrolled in our study showed that PM_2.5_ exposures averaged 43 μg/m^3^, with a range of 10.3–147.3 μg/m^3^. The mean weight, length, HC at birth, and gestational age for infants under study were 3439.8 g, 54.6 cm, 33.9 cm, and 39.5 weeks, respectively. The newborns of mothers with higher exposures to fine particles in the period of monitoring (above the median of 36.3 μg/m^3^) showed shorter length at birth by 0.9 cm. The corresponding reductions in HC and birth weight were 0.3 cm and 128.3 g, respectively (Table 1).

**Figure 1:**
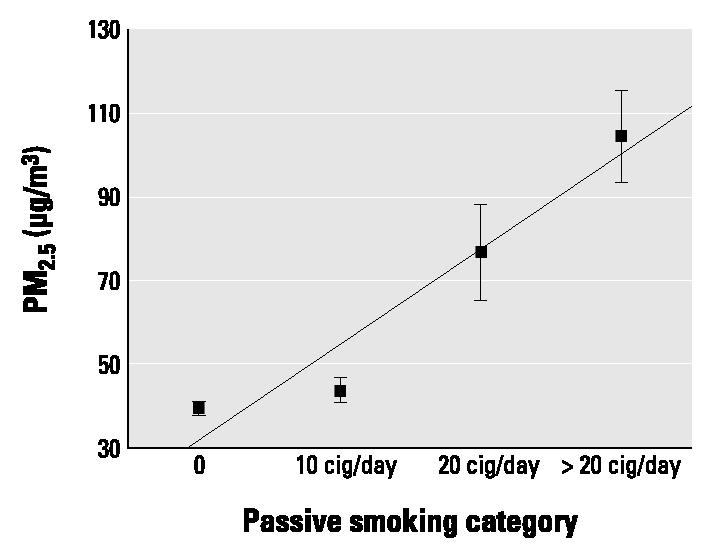
Personal PM_2.5_ level by passive smoking category [cigarettes (cig)/day]. Data are mean ± SE.

In the subsequent statistical analysis of the data, we used multiple linear multivariate regression models to examine the relationship between birth outcomes and the effect of fine particles and ETS. For each birth outcome, we constructed a separate model where dependent variables included gestational age, sex of child, season of birth, and variables on quality of air (ETS and level of PM_2.5_), as well as the anthropometry of the mother. Both variables of air quality correlated significantly (*p* = 0.01) with each other (*r* = 0.35), and the level of personal exposure to PM_2.5_ depended on the number of cigarettes smoked daily at home (Figure 1).

Tables 2–4 present the standardized β regression coefficient of PM_2.5_ on the birth outcomes after accounting for all dependent variables. The regression model explained 33.4% of the variability in birth weight (β = −200.8, *p* = 0.03), and both regression coefficients for PM_2.5_ and birth length (β = −1.44, *p* = 0.01) and HC (β = −0.73, 0.02) were significant as well. In all regression models the effect of ETS was insignificant. Predicted reduction in birth weight at an increase of exposure from 10 to 50 μg/m^3^ was 140.3 g. The corresponding predicted reduction of birth length would be 1.0 cm, and of HC, 0.5 cm.

Finally, we explored the hypothesis that PM_2.5_ had an adverse effect on gestational age. We did not find in the linear multivariate regression model that PM_2.5_ or ETS shortened the duration of pregnancy.

## Discussion

Until now, there have been no studies of effects of personal exposure to fine particles on reproductive health and birth outcomes. Our study draws attention to the fact that not only lower birth weight but also reduction in length and HC at birth might be caused by prenatal exposure to pollutants during pregnancy.

Analysis of personal air samples from the pregnant women enrolled in the Krakow study showed that total personal PM_2.5_ exposures averaged 43.1 μg/m^3^ with a range of 10.3–147.3 μg/m. None of the women under study in Krakow reported heavy exposure to dusty environments in the working hours. The PM_2.5_ level in the Krakow study was very high and had a wide range of exposure, compared with data from the United States, where the range of annual mean for PM_2.5_ measured in various sites is 1.2–14.2 μg/m^3^ (U.S. EPA 2003). However, the PM_2.5_ exposure observed in Krakow would be comparable with that observed in the Czech Republic, where the daily mean is 35.6 μg/m^3^ (Dejmek et al. 1999).

The analysis of birth outcomes indicated a significant inverse correlation between concentrations of fine particles and fetal growth. The adjusted effect of exposure to PM_2.5_ was reflected in significantly lower mean weight and length at birth and lower mean HC of newborns. The newborns of mothers exposed to higher concentrations of fine particles (above the median of 36.3 μg/m^3^) showed shorter length at birth by 0.9 cm. The corresponding reductions in HC and birth weight were 0.3 cm and 128.3 g. We estimated from the regression equations that an increase of exposure from 10 to 50 μg/m^3^ of fine particles would reduce length at birth by 1.0 cm. The corresponding reductions of HC and birth weight would be 0.5 cm and 140.3 g, respectively.

Our study showed a significant positive interrelationship between self-reported ETS level and total personal exposure to PM_2.5_, which to a great extent depended on the number of reported cigarettes smoked daily at home. This interrelationship creates difficulties in separating the effect of ETS on the birth outcomes from that attributed to fine particles. However, none of the models with both ETS and PM_2.5_ showed a significant effect of ETS. Moreover, stepwise regression indicated that adding the ETS variable into the model did not explain better the amount of variability in birth outcomes due to air contamination. Therefore, we think that the effect of ETS confirmed in many previous studies may result from its interrelationship with PM_2.5_. In studies where the birth outcomes were not controlled by the PM_2.5_ level, the effect of ETS could be demonstrated.

The biologic mechanisms whereby PM_2.5_ might cause adverse pregnancy outcomes are unclear. PM_2.5_ might be a proxy measure of a whole complex of toxic agents present in the environment—including PAHs—that could adversely affect fetal growth. It is well known that fine particles are virtually always present in particle-generating processes, especially combustion processes that generate other toxic agents as well. Typically, the ambient fine particle fraction contains constituents of tobacco and wood smoke, organic compounds, sulfates, metals, and soot (Spengler et al. 2001). Therefore, it would be reasonable to assume that PM_2.5_ represents a wide spectrum of environmental hazards that may be implicated in intrauterine fetal growth. Air pollutants may affect DNA, as evidenced by observations that placental DNA adducts are more common in areas with higher levels of pollution (Topinka et al. 1997) and that altered fetal growth has been associated with PAH–DNA adducts (Perera et al. 1998).

Our data indicating that personal exposure to fine particles has a stronger relationship with birth outcomes than does ETS may result from the fact that the measurement of ETS exposure based on interviews with pregnant women could be biased. If respondents underestimated their ETS exposure, then its effect may appear much weaker in comparison with the objective measurements of fine particles. However, the level of fine particles is the function not only of ETS, which is the major source of indoor pollution, but also of PM generated from other sources such as fossil fuel combustion.

Another potential limitation of our study comes from the fact that personal monitoring of exposure to fine particles among pregnant women was performed over the short period of 48 hr in the second trimester of pregnancy. However, to evaluate the correlation between the level of PM_2.5_ measured over 48 hr in the second trimester of pregnancy with those in the second and the third trimesters, a series of repeated measurements in each trimester was carried out in the subsample of 51 pregnant women who were recruited in the first trimester. The concentration of PM_2.5_ (mean ± SD) in the second trimester was 44.4 ± 46.5 μg/m^3^, but it was not significantly different from the mean concentration in the first trimester (46.2 ± 34.0 μg/m^3^) or in the third trimester (35.9 ± 35.3 μg/m^3^). The latter results suggest that the mean levels of fine particles were rather stable over the whole pregnancy. This provides some confidence that the measurements of personal level of exposure to fine particles taken in the second trimester may also be representative for other pregnancy periods.

**Figure 2:**
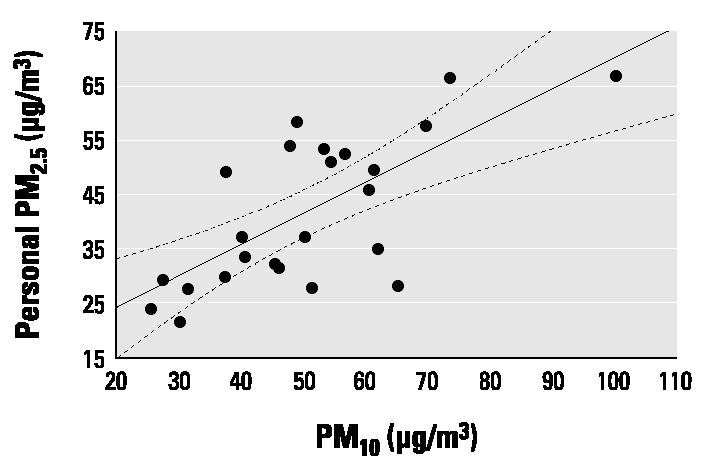
Correlation between mean monthly personal PM_2.5_ measurements and mean monthly PM_10_ concentrations from areawide ambient monitoring (circles). Solid line, regression; dashed lines, 95% CI.

We could also demonstrate that total personal exposure to PM_2.5_ measured over 48 hr correlated well with the PM_10_ concentrations obtained from the monitors of the municipal air pollution network of Krakow, which were located in the residence areas of the subjects under study (Figure 2). We observed consistency between monthly means of PM_10_ measured by the local ambient monitors and the monthly means of total personal exposure to fine particles measured over 48 hr in the second trimester. This suggests that the extrapolation of ambient measurements to personal exposure may be reasonably approximated. However, the extent to which the ambient measurements reflect the individual exposure level may be different in various populations. First, it would depend largely on the quality of the ambient network of air pollution stations and its appropriate coverage of the given residency areas. Besides different lifestyles and mobility of women over the study period, substantial seasonal changes in air pollution due to weather and meteorologic conditions may be significant.

In our study, the most important confounders of the birth outcomes such as the presence of chronic diseases or active tobacco smoking by mothers in pregnancy have been removed through entry criteria. Other risk factors thought to affect the probability of delivery of newborns with lower growth, such as maternal height or prepregnancy weight, gestation age, sex of child, and season of birth, were also accounted for in the analysis.

Over the last decades there has been growing concern over the health effects associated with air pollution. The studies were concerned mainly with morbidity and mortality from respiratory diseases, occurrence of respiratory symptoms, pulmonary function, and physician office visits. To date, there have been a limited number of studies investigating the association between air pollution and adverse birth outcomes, and the conclusions were somewhat inconsistent. A study conducted in China suggested that exposure to TSP and SO_2_ was associated with an excess risk of preterm delivery (Xu et al. 1995; Yang et al. 2002) and LBW (Wang et al. 1997). Several studies observed an association of TSP and SO_2_ with LBW and found increased risk of IUGR in a highly polluted region in the Czech Republic (Bobak and Leon 1999; Dejmek et al. 1999, 2000; Sram 1999). Ritz and Yu (1999) found that high concentrations of CO and PM_10_ during the last trimester of pregnancy may increase the risk of LBW for term babies. Our prior study also showed that, after controlling for dietary and smoking sources of the pollutants, PAH–DNA adducts in cord blood were inversely associated with birth weight, length, and HC (Perera et al. 1998). In contrast, in a study performed in southern Sweden, Landgren (1996) could not confirm the hypothesis that air pollution affected the incidence of short gestation and LBW. Some researchers (Bhopal et al. 1994; Smrcka and Leznarova 1998) found no association between either residential proximity to a coking plant or major steel and petrochemical industries and birth outcomes in the United Kingdom. The study period, size of the population, and number of cases were large. However, Axelsson and Molin (1988) found that the miscarriage rate was slightly elevated in areas exposed to emissions from petrochemical industries. None of the studies used personal monitors of PM_2.5_ in the assessment of exposure.

The results of our study are of public concern because adverse birth outcomes have been associated in other studies with more health problems and reduced cognitive development in childhood (Perera et al. 2002). Follow-up of the cohort will permit determination of the longer-term sequels of prenatal exposures and adverse birth outcomes. The results of our study may have implications not only for the health and development of children but also for adult health. Epidemiologic studies in children indicate that prenatal hazards that restrict fetal growth may be associated with small but measurable delays in motor and social development through childhood and reduced cognitive development (Bacharach and Baumeister 1998; Hack et al. 1991; Hediger et al. 2002; Saigal 2000). There is also evidence of associations between birth size and future development of adult diseases, such as type 2 diabetes and coronary artery disease (Godfrey and Barker 2001; Phillips 2000). It is believed that these associations arise as a result of the phenomenon of “programming,” which involves persisting changes in structure and function caused by environmental factors during critical and vulnerable periods of early development. However, other explanations, including the operation of genetic factors and programming of certain endocrine axes, have also been suggested to explain this observation.

Our study provides convincing epidemiologic evidence based on a cohort that prenatal exposure resulting from high personal maternal exposure to fine particles is associated with adverse effects on the developing fetus. These results indicate the need to reduce ambient fine particulate concentrations. However, further research should help establish possible biologic mechanisms explaining the observed relationship.

## Figures and Tables

**Table 1:** Characteristics of the study sample by PM_2.5_ level of personal exposure during pregnancy (mean ± SD).

Variable	Low level*^a^* (*n* = 180)	High level*^b^* (*n* = 182)
Mother’s age	28.1 ± 3.4	28.1 ± 3.9
Mother’s height (cm)	164.7 ± 5.3	165.3 ± 6.0
Mother’s weight (kg)	58.6 ± 10.2	58.3 ± 7.6
Gestational age (weeks)	39.6 ± 1.3	39.4 ± 1.4
Length at birth (cm)	55.1 ± 2.7*	54.2 ± 2.6
Birth weight (g)	3504.3 ± 471.1*	3376.0 ± 453.1
HC at birth (cm)	34.1 ± 1.5*	33.8 ± 1.4

a Low level, ≤36.3 μg/m^3^.

b High level, > 36.3 μg/m^3^.

* Significantly higher (analysis of variance, *p* < 0.05) compared with the group with higher exposure to PM_2.5._

**Table 2:** Regression summary of dependent variable (birth weight) on log PM_2.5_ exposure and confounding variables (number of pregnancies, height and prepregnancy weight of mother, sex of newborn, and gestational age).*^a^*

Variable	Coefficient	95% CI	*t*-Value	*p*-Value
Intercept	−4876.72			
Education (years of schooling)	10.886	−3.814 to 25.586	1.48	0.14
No. of pregnancies	61.451	13.121 to 109.781	2.54	0.01
Maternal height (cm)	10.958	3.278 to 18.638	2.85	0.00
Prepregnancy weight (kg)	9.743	4.867 to 14.619	4.00	0.00
Gestational age (weeks)	160.288	130.575 to 190.001	10.79	0.00
Sex of child	−212.802	−293.914 to −131.691	−5.25	0.00
Season				
Autumn	−63.658	−182.217 to 54.900	−1.07	0.28
Winter	49.179	−67.036 to 165.395	0.85	0.40
Spring	23.544	−92.276 to 139.363	0.41	0.68
Log PM_2.5_	−200.821	−385.968 to −15.674	−2.17	0.03
ETS	32.008	−91.554 to 155.569	0.52	0.60

CI, confidence interval.

a *R* = 0.588; *R*^2^ = 0.334.

**Table 3:** Regression summary of dependent variable (length at birth) on log PM_2.5_ exposure (continuous) and confounding variables (number of pregnancies, height and prepregnancy weight of mother, sex of newborn, and gestational age).*^a^*

Variable	Coefficient	95% CI	*t*-Value	*p*-Value
Intercept	13.8235			
Education (years of schooling)	0.021	−0.070 to 0.111	0.45	0.65
No. of pregnancies	0.267	−0.032 to 0.566	1.79	0.07
Maternal height (cm)	0.067	0.020 to 0.115	2.83	0.00
Prepregnancy weight (kg)	0.040	0.010 to 0.070	2.66	0.01
Gestational age (weeks)	0.792	0.608 to 0.975	8.62	0.00
Sex of child	−1.150	−1.652 to −0.649	−4.59	0.00
Season				
Autumn	−0.464	−1.197 to 0.269	−1.27	0.21
Winter	0.068	−0.650 to 0.787	0.19	0.85
Spring	−0.063	−0.779 to 0.654	−0.17	0.86
Log PM_2.5_	−1.439	−2.583 to −0.294	−2.51	0.01
ETS	−0.244	−1.008 to 0.520	−0.64	0.52

CI, confidence interval.

a *R* = 0.518; *R*^2^ = 0.262.

**Table 4:** Regression summary of dependent variable (HC at birth) on log PM_2.5_ exposure (continuous) and confounding variables (number of pregnancies, height and prepregnancy weight of mother, sex of newborn, and gestational age).*^a^*

Variable	Coefficient	95% CI	*t*-Value	*p*-Value
Intercept	17.2662			
Education (years of schooling)	0.043	−0.006 to 0.092	1.76	0.08
No. of pregnancies	0.186	0.025 to 0.347	2.31	0.02
Maternal height (cm)	0.028	0.002 to 0.053	2.17	0.03
Prepregnancy weight (kg)	0.026	0.009 to 0.042	3.14	0.00
Gestational age (weeks)	0.301	0.202 to 0.400	6.08	0.00
Sex of child	−0.782	−1.052 to −0.511	−5.78	0.00
Season				
Autumn	−0.020	−0.416 to 0.375	−0.10	0.92
Winter	0.111	−0.277 to 0.498	0.57	0.57
Spring	0.134	−0.253 to 0.520	0.69	0.49
Log PM_2.5_	−0.729	−1.347 to −0.112	−2.36	0.02
ETS	0.151	−0.261 to 0.563	0.73	0.46

CI, confidence interval.

a *R* = 0.472; *R*^2^ = 0.214.
